# Secondhand Smoke Exposure Timing Triggers Distinct Placental Responses in Mouse Pregnancy

**DOI:** 10.3390/cells14211735

**Published:** 2025-11-05

**Authors:** Archarlie Chou, Ethan Frank, Matt Reall, Olivia Hiatt, Logan Beck, Paul R. Reynolds, Brett E. Pickett, Juan A. Arroyo

**Affiliations:** 1 Department of Microbiology and Molecular Biology, Brigham Young University, Provo, UT 84602, USA; 2 Department of Cell Biology and Physiology, Brigham Young University, 3052 LSB, Provo, UT 84602, USA

**Keywords:** secondhand smoke, preeclampsia, intrauterine growth restriction, placental dysfunction, RNA-sequencing

## Abstract

Secondhand smoke (SHS), found in about 57.6% of global public areas as a widespread environmental hazard, has been associated with negative effects during pregnancy, such as preeclampsia (PE) and intrauterine growth restriction (IUGR). Our research investigated the impact of SHS on placental issues in a C57BL/6 model that simulates PE and IUGR in mice. We administered SHS to pregnant mice through a nose-only delivery method, beginning either on embryonic day 12.5 (prior to spiral artery (SA) invasion; labeled SHS-6D) or day 14.5 (following SA invasion; labeled SHS-4D), continuing up to E18.5. Control animals received only ambient air. We employed bulk RNA sequencing to assess and describe changes in placental gene expression patterns. For the SHS-4D group, which mimicked IUGR, compared to untreated controls, results showed elevated levels of inflammation-related genes (IL11RA, CHI3L1) alongside likely interference in pathways for antibody-triggered complement activation, marked by reduced expression of C1QA, C1QB, and C1QC. Immune profiling also indicated decreased macrophage activity in the placentas of the SHS-4D group relative to those from normal pregnancies at term. In contrast, the SHS-6D versus control analysis revealed lowered expression of collagen-related genes (COL1A1, COL4A5, COL4A6, COL17A1). Additionally, SHS-6D exhibited higher levels of genes associated with cell-based lysis processes compared to SHS-4D. An evaluation of the existing literature revealed that nearly every differentially expressed gene (DEG) identified in our work has been reported in studies associated with SHS exposure. Yet, few of these DEGs are discussed alongside PE or IUGR in prior reports, highlighting gaps in knowledge about how SHS triggers these conditions. Overall, we determined that the timing of SHS exposure in pregnant mice results in unique patterns of gene regulation and involvement in biological pathways.

## 1. Introduction

Secondhand smoke (SHS), defined as the unintended breathing in of smoke from tobacco products, represents a widespread environmental hazard that carries major consequences for public health, especially within less developed regions [1]. This type of smoke includes no fewer than 250 substances known to be harmful or cancer-causing, with elements like nicotine, carbon monoxide, and polycyclic aromatic hydrocarbons (PAHs) that are capable of passing through the placenta during gestation and interfering with the growth of the fetus [1,2,3]. Current research highlights the dangers posed by various environmental factors, SHS among them, which can worsen oxidative damage and inflammatory responses in the placenta. As an example, PAHs are associated with heightened levels of DNA oxidation and breakdown of lipids in placental structures [4]. In a comparable way, vapors from electronic cigarettes—which overlap in some of their chemical makeup with SHS—trigger oxidative damage and inflammation within the placenta, leading to negative effects on the fetus [5]. Moreover, contaminants in the air that resemble parts of SHS raise levels of placental indicators for oxidative and nitrosative damage, including 8-OHdG and 3-NTp, reinforcing the connection between such exposures and issues arising during gestation [6].

Factors from the environment, such as smoking by the mother, polluted air, and dietary habits, are key players in unfavorable results during pregnancy like preeclampsia (PE) and intrauterine growth restriction (IUGR). These conditions significantly drive rates of illness and death for mothers and newborns around the world, emphasizing why examining how SHS influences placental processes is vital for public health efforts [7,8,9].

PE constitutes a disorder involving high blood pressure during gestation, impacting roughly 2–8% of pregnancies on a global scale. It features the emergence of elevated blood pressure beyond 20 weeks in someone who was previously without hypertension, combined with protein in the urine or dysfunction in organs or the uteroplacental system, potentially accompanied by swelling. When advanced, PE may evolve into eclampsia, a dangerous state marked by convulsions that endangers life [10,11,12,13]. Furthermore, PE is responsible for more than 45,000 deaths among mothers each year [13]. The condition arises from inadequate formation of the placenta, limited penetration by trophoblasts, and insufficient remodeling of spiral arteries (SA), which cause diminished blood flow to the placenta and widespread issues with maternal blood vessel linings [14,15]. Still, the core reasons remain unclear because of their complex and varied physiological origins [16].

IUGR, a connected condition during pregnancy, refers to a fetus weighing less than the 10th percentile based on its age in weeks [17]. Globally, IUGR occurs in 3–10% of gestations, with figures climbing to 20–30% in nations that are developing [17,18]. Multiple elements contribute to its causes, encompassing traits of the mother like ethnicity, duration of pregnancy, and her own weight at birth, though the primary factor is how well the uterus supplies blood [19]. Exposure to SHS plays a direct part in causing IUGR through harm to placental growth. Studies using mice show that SHS leads to decreased weights in both fetuses and placentas, with notable impacts appearing after just four days [5]. Such reductions in growth occur due to placental malfunctions, which involve greater cell death, imbalanced proteins that control development, and suppressed mTOR activity in the placenta—a key controller for trophoblast penetration and the movement of nutrients [20]. The underlying cellular processes for placental issues caused by SHS go beyond this, including heightened oxidative damage, harm to genetic material, and disrupted control of inflammation. Additionally, SHS interferes with how the placenta manages immunity by changing cytokine levels and other immune regulators, resulting in ongoing inflammation and poor adjustment at the boundary between mother and fetus [21]. These alterations in immunity and cellular function are worsened by problems with blood vessel linings and flaws in restructuring the extracellular matrix (ECM), which hinder blood vessel formation in the placenta and the provision of essential nutrients. Together, these mechanisms create a clear connection linking SHS exposure to IUGR and associated issues like PE [22].

In addition to those processes, the classical complement pathway activated by antibodies has a notable influence on IUGR. Through this pathway, C1Q produces anaphylatoxins C3A and C5A, which may draw in white blood cells, upset the equilibrium of blood vessel formation by boosting the anti-angiogenic factor sFlt-1, and hinder the movement of trophoblasts along with the restructuring of spiral arteries—all of which limit blood supply and restrict how the fetus grows [23,24]. Moreover, activating the complement system might result in loss of the fetus and slowed growth, highlighting the dangers it poses in gestation [25]. Apart from this activation, lymphocytes in the decidua during PE display greater levels of destructive agents like granzyme K, FasL, and interferon-γ, indicating elevated immune responses that could lead to a lack of tolerance in the immune system [26].

This research sought to employ a C57BL/6 model in mice alongside advanced bulk RNA sequencing methods to detail the gene expression changes prompted by SHS at different stages of gestation. In particular, it represents among the initial efforts employing a nose-only method for exposure to explore the impact of SHS timing—whether prior to or following spiral artery invasion in development—on patterns of gene activity and related biological routes critical for placental operations, especially regarding the onset of PE or IUGR, as well as overall placental performance. The approach we used for SHS mimics exposure to tobacco smoke in the environment, setting it apart from direct smoking [27]. Such a difference matters because SHS might miss out on the filtering of air and certain combustion aspects that could offer physiological safeguards in active smoking, yet it continues to provoke inflammatory effects. The results from our work shed light on how the duration of contact with SHS—a typical toxic substance in surroundings—affects the well-being of the placenta, delivering key understanding of its contribution to poor outcomes in pregnancy.

## 2. Materials and Methods

### 2.1. Animal Husbandry and Exposure Protocol

**Figure 1:**
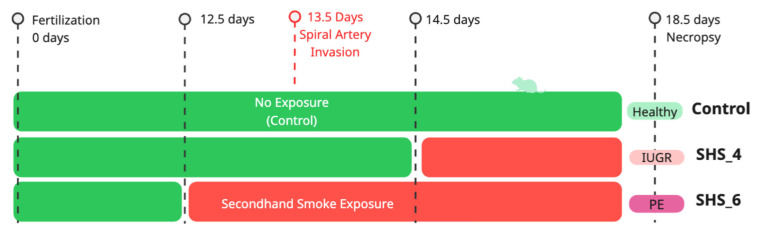
Experiment Timeline and Design of SHS Exposure.

Mice of the C57BL/6 strain were acquired from Jackson Laboratory (Bar Harbor, ME, USA) and kept under regulated conditions featuring a 12 h alternation between light and darkness, along with unrestricted availability of regular diet and drinking water. After verifying pregnancy, the animals were exposed to SHS through the InExpose nose-only apparatus (Scireq, based in Montreal, Canada) (Figure 1). The initiation of exposures occurred at embryonic day 12.5 (E12.5, prior to the entry of trophoblasts into spiral arteries) or at E14.5 (following the commencement of SA invasion), extending to E18.5 in each case. Although certain investigations note uNK cell-facilitated SA breakdown occurring as soon as E9–10, ahead of trophoblast participation, trophoblast entry in murine models tends to be less extensive and more confined temporally relative to human cases, typically maximizing from E10.5 to E14 [28,29]. In response to this diversity, our definition of ‘pre-invasion’ in the C57BL/6 framework is set at E12.5, denoting the transitional stage for this investigation. Regarding SHS, the apparatus administered a solitary puff each minute throughout a 10 min duration. These exposure settings drew from previous research to confirm tolerability for the animals and to attain particle densities within permissible boundaries. The mice were allocated to three categories (n = 6 in each): a Control category (ambient room air, Control), a group with SHS for 6 days (SHS-6D), and one with SHS for 4 days (SHS-4D). Necropsies took place at E18.5, during which we documented weights for fetuses and placentas. We obtained urine specimens for proteinuria evaluation and promptly preserved placental samples in liquid nitrogen for later examination of proteins.

### 2.2. Evaluation of Blood Pressure

Non-invasive measurements of systolic and diastolic blood pressure utilized the CODA tail-cuff apparatus (Kent Scientific, Torrington, CT, USA), complete with an automatic occlusion cuff and heating platform. Readings occurred on every alternate day, commencing from the initial exposure date, distinctly from E12.5 (for SHS-6D) or E14.5 (for SHS-4D), extending to the E18.5 necropsy. All groups had blood pressure assessments conducted before exposure began. The mice underwent mild restraint within a transparent cylindrical holder of medium dimensions equipped with an adaptable head restraint (Kent Scientific) for roughly five minutes per session. Recordings were made on a preheated platform (30–32 °C) to lessen any distress.

### 2.3. Assessment of Proteinuria

For gauging proteinuria as a sign of PE, urine from mice was gathered during necropsy and examined via a colorimetric strip approach. Results from the strips were classified as negative, trace, +1 (30 mg/dL), +2 (100 mg/dL), +3 (300 mg/dL), or +4 (≥2000 mg/dL), where +3 and +4 denote substantial proteinuria aligning with PE. To quantitatively verify these outcomes, we applied an Albuminuria Fluorometric Assay Kit (MyBioSource, San Diego, CA, USA) to substantiate the strip observations.

### 2.4. RNA Isolation, Preparation of Libraries, and Sequencing Process

Extraction of total RNA from placental tissues of the mice employed the Direct-zol RNA MiniPrep Plus Kit combined with TRI Reagent (Zymo Research, Irvine, CA, USA, Cat No. R2052). Samples from placentas were broken down in TRI Reagent at peak velocity, then centrifuged to isolate the supernatant. This supernatant was combined with an equivalent amount of pure ethanol and applied to a Zymo-Spin IICR column. Treatment with DNase I was performed according to the producer’s directions, followed by subsequent washes on the column as specified. Elution of RNA occurred in 30 μL of water free from DNase/RNase, followed by storage at −80 °C pending additional steps. Library construction involved separating mRNA via magnetic beads conjugated to poly-dT oligos for cDNA production. Synthesis of first-strand cDNA used random hexamer primers, followed by second-strand creation, polishing of ends, addition of A-tails, and attachment of Illumina adapters. The libraries experienced selection for size, amplification, and cleansing procedures. Quantification of libraries was performed using a Qubit fluorometer (Thermo Fisher Scientific, Waltham, MA, USA), in conjunction with qPCR, while assessment of size distribution relied on a Bioanalyzer (Agilent Technologies, Santa Clara, CA, USA). Sequencing in paired-end format for the barcoded, pooled, and normalized libraries took place on an Illumina NovaSeq platform (Novogene, Sacramento, CA, USA).

### 2.5. Analysis of RNA-Seq Data

Preprocessing for the bulk RNA-sequencing data utilized the RASflow workflow [30]. In summary, this process automates quality assessment via FastQC [31], adapter removal through TrimGalore, alignment and counting with Salmon, aggregation of transcripts to genes using tximport, and determination of differential expression via edgeR (v4.6.2) across three contrasts: (1) SHS-4D against mock-treated controls; (2) SHS-6D against mock-treated controls; and (3) SHS-6D against SHS-4D [30]. Genes deemed significantly differentially expressed were those with an FDR-adjusted *p*-value below 0.05. Enrichment of pathways applied the signaling pathway impact analysis (SPIA) method (V2.60.0) [32] from the EnrichmentBrowser R Bioconductor package (V2.38.1), drawing from KEGG [33] and Reactome resources [34]. In addition, Gene Set Enrichment Analysis (GSEA; V1.70.1) served for pathway enrichment, applying a *q*-value cutoff under 0.05 [35,36]. Subsequent drug target evaluation used the Pathway2Targets method [37,38]. Human equivalents of the differentially expressed mouse genes were utilized.

Heatmaps enhanced with pathway data were created from the notably enriched pathways per condition. Even though pathways encompass numerous genes, visualizations focused solely on DEGs in significant pathways for enhanced readability across all heatmaps.

Analysis of gene associations employed a tailored Python script (V3.11.13) leveraging PubTator APIs for literature queries [39]. Queries were formed as “@DISEASE_Pre_Eclampsia”, “@DISEASE_Fetal_Growth_Retardation”, and “secondhand smoke” per gene. The count of retrieved references per query estimated the robustness of associations between terms and genes in the literature.

Deconvolution for immune cells used CibertsortX [38], facilitating comparisons of immune signals within bulk RNA-seq data. The signature matrix for mouse cells was acquired [40]. Application of the raw count matrix occurred through the web platform. Corrections for B-mode batches and 500 permutations were executed with the raw count matrix alongside the mouse cell signature matrix [40]. Adjustments for FDR (BH) appeared on boxplots, accounting for displayed cell type counts.

### 2.6. Statistics

To ascertain statistical differences in fetal and placental weights, proteinuria levels, and blood pressure values, we used an unpaired Student’s t-test, establishing significance at *p* < 0.05. Regarding the RNA-sequencing information, edgeR handled differential gene expression analysis via a negative binomial model, with multiple comparison adjustments via Benjamini–Hochberg FDR (BH-FDR) method (FDR < 0.05).

## 3. Results

### 3.1. Blood Pressure, Proteinuria, and Fetal and Placental Weights

**Figure 2:**
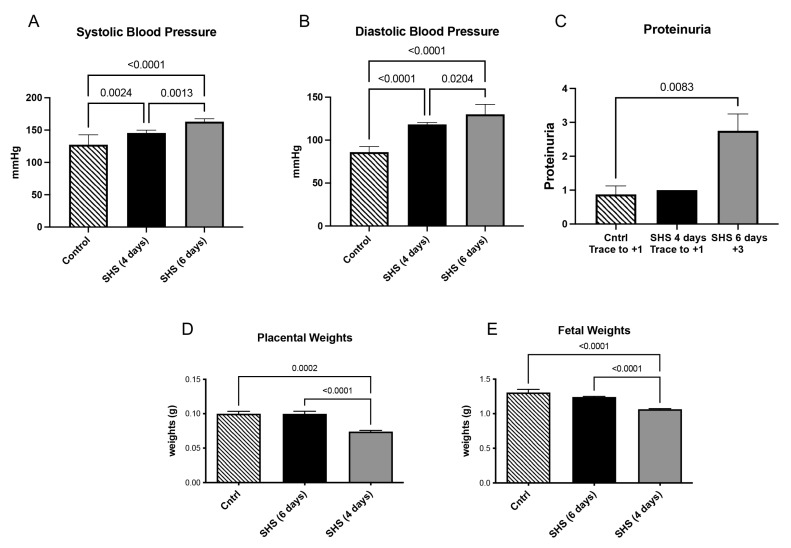
Effects of SHS exposure on physiological parameters (mean ± SD, n = 6 per group). (**A**) Systolic and (**B**) diastolic blood pressure were increased by SHS at all time periods studied, (**C**) Proteinuria was increased only with 6 days of SHS treatment, (**D**) placental and (**E**) fetal weights were decreased with 4 days of SHS treatment.

Consistent with our hypothesis that SHS timing differentially affects pregnancy outcomes, we first evaluated physiological markers of PE (hypertension, proteinuria) and IUGR (reduced weights). Increased blood pressure is a hallmark of PE. To determine if blood pressure changed after SHS treatment, daily blood pressure measurements were taken. At the time of necropsy (E18.5), both systolic and diastolic blood pressures were significantly increased following four days (*p* < 0.003) or six days (*p* < 0.03) with SHS treatment (Figure 2A–C). Increased proteinuria is another marker for complications with PE. We observed a significant increase in proteinuria (2.5-fold increase; *p* < 0.004) within six days of SHS treatment in our samples (Figure 2D). A hallmark of IUGR development is the decreased weight of both the placenta and the fetus. We then determined the changes in placental and fetal weight during SHS treatment. We found that both placental (1.4-fold; *p* < 0.0001) and fetal weight (1.4-fold; *p* < 0.001) were significantly decreased following four days of SHS treatment (Figure 2D,E). In contrast, no significant differences in placental and fetal weights were observed after six days of SHS treatment (Figure 2D,E). These explicit changes, including a 2.5-fold increase in proteinuria in SHS-6D (*p* < 0.004) and 1.4-fold reductions in placental/fetal weights in SHS-4D (*p* < 0.0001 and *p* < 0.001, respectively), directly support the temporal hypothesis, with SHS-6D inducing PE-like symptoms and SHS-4D driving IUGR-like growth restrictions.

To address the hypothesis of timing-dependent gene regulation, we identified differentially expressed genes (DEGs) across comparisons, revealing distinct patterns in immune, extracellular, and PE/IUGR-related functions. To investigate the gene regulatory effects of SHS after SA invasion, we examined the top five genes in the SHS-4D versus control comparison with the highest log_2_-fold changes in each direction (Table 1). These genes were associated with immune responses, extracellular structure organization, and cellular detoxification.

Within the immune response category, *IL11RA*, a mediator of inflammatory signaling, and *CHI3L1*, an established marker of inflammation, were both upregulated. In the extracellular structure category, *UPK3BL*, a structural protein of urothelial plaques; *ARHGAP40*, predicted to participate in actin filament organization; and *KRT15*, an intermediate filament protein, were downregulated.

In the detoxification category, *UGT1A9*, a UDP-glucuronosyltransferase involved in xenobiotic metabolism, was significantly downregulated. Finally, *ECE2*, which encodes endothelin-converting enzyme 2, was upregulated; this enzyme participates in the processing of vasoactive peptides within endothelial cells.

To identify genes associated with PE during SHS exposure, we analyzed the top DEGs in the SHS-6D versus control comparison (Table 2), revealing functional patterns linked to inflammation, extracellular structure regulation, and PE pathogenesis again.

In the immune response category, *TPSAB1*, a serine protease expressed in mast cells, and *CD6*, a T-cell costimulatory receptor, were upregulated, while *MUC16*, a key component of mucous protective barriers, was downregulated. In the extracellular structure category, *UPK3BL*, a structural protein of the urothelial surface, and *KRT5*, an epithelial cytoskeleton component, were both downregulated. Among genes directly associated with PE, *EDN2*, encoding endothelin-2, a potent vasoconstrictor peptide, was significantly upregulated.

We investigated relevant genes by analyzing the SHS-6D vs. SHS-4D comparison (Table 3). We found that the SHS-6D timepoint included upregulated *GZMB*, *GZMD*, and *PRF1* compared to the SHS-4D. *PRF1*, perforin 1, is a pore-forming antimicrobial peptide, while *GZMB* (granzyme B) and *GZMD* (granzyme D) are recognized as key mediators of cell-mediated cytotoxicity via the release of cytotoxic granules that induce target cell death. Collectively, these DEGs (FDR < 0.05 across tables) demonstrate distinctions that reflect our hypothesis: immune/extracellular dysregulation in SHS-4D vs. vasoconstriction/ECM changes in SHS-6D.

### 3.2. Pathway Enrichment Results

**Figure 3:**
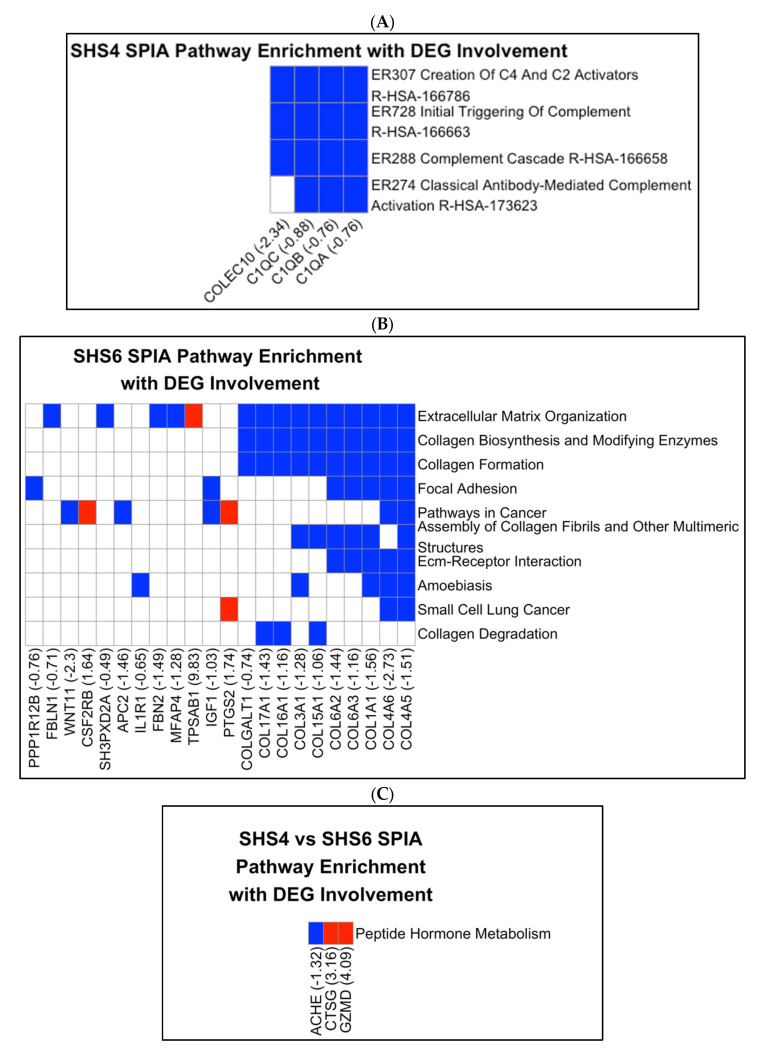
SPIA Pathway Enrichment with DEG. The *y*-axis displays the names of significantly enriched pathways from KEGG and Reactome (SPIA, FDR-adjusted *p*-value < 0.05), sorted by the number of genes they contain. The *x*-axis displays the gene symbols and log_2_ fold change (log_2_FC) values for differentially expressed genes (DEGs, FDR < 0.05), sorted by pathway occurrence. Tile colors indicate upregulated (red) or downregulated (blue) gene regulation, and white for absent genes. (**A**) SHS-4D vs. mock-treated controls, (**B**) SHS-6D vs. mock-treated controls, (**C**) SHS-6D vs. SHS-4D.

We then performed pathway enrichment analyses to further calculate the enriched KEGG/Reactome pathways (SPIA, pGWER < 0.05) that were unique to each exposure timepoint. We next analyzed each list of DEGs to determine the significant pathways among SHS-4D-induced IUGR (SHS-4D vs. mock-treated controls) and SHS-6D-induced PE (SHS-6D vs. mock-treated controls) phenotypes. In the SHS-4D vs. mock-treated controls comparison (Figure 3A), we identified pathways related to the innate immune response, such as the creation of C4 and C5 activators, initial triggering of complement, complement cascade, and classical antibody-mediated complement activation, which were enriched and predicted to be downregulated based on the SPIA topological algorithm. *COLEC10* (log_2_FC = 2.34; FDR = 0.015), *C1QA* (log_2_FC = −0.764; FDR = 0.016), *C1QB* (log_2_FC = −0.761; FDR = 0.003), and *C1QC* (log_2_FC = −0.879; FDR = 0.005). Downregulation of complement-related genes, such as *C1QA* (log_2_FC = −0.764; FDR = 0.016), explicitly drives the predicted inhibition.

Furthermore, in the SHS-6D vs. mock-treated controls comparison (Figure 3B), pathways associated with the ECM are enriched. These pathways include ECM organization, collagen biosynthesis, modification by enzymes, collagen formation, focal adhesion, assembly of collagen fibrils, ECM-receptor interaction, and collagen degradation. Genes involved were overwhelmingly downregulated. Interestingly, pathways in cancer, amebiasis, and small-cell lung cancer are also enriched. The observed pathway enrichment was driven in part by the upregulation of *PTGS2* (log_2_FC = 1.74; FDR = 0.003), part of the pathway gene set. *PTGS2* encodes prostaglandin-endoperoxide synthase 2, an enzyme that exhibits both peroxidase and dioxygenase activities.

In the SHS-6D vs. SHS-4D comparison (Figure 3C), only the peptide hormone metabolism pathway was significantly enriched due to the upregulation of *GZMD* (log_2_FC = 4.09; 5.16 × 10^−6^) and *CTSG* (log_2_FC = 3.16; FDR = 8.51 × 10^−4^), and the downregulation of *ACHE* (log_2_FC = −1.32; FDR = 0.0018). *CTSG*, Cathepsin G, is found in granules and may participate in the engulfment and killing of pathogens, as well as connective tissue remodeling. These enrichments directly address the hypothesis, confirming distinct etiologies: complement inhibition in SHS-4D/IUGR vs. collagen/ECM downregulation in SHS-6D/PE.

### 3.3. Drug Target Analysis

Following the results of the pathway analysis, specifically the disruption of the complement pathway in SHS-4D vs. mock-treated controls and the disruption of the collagen-forming pathway in SHS-6D vs. mock-treated controls, we decided to investigate current drugs that could reverse these disruptions. However, upon close examination of the effects of downregulation of *C1QA*, *C1QB*, *C1QC*, and *COLEC10* on the complement activation pathways on Reactome, we realized that these proteins act intercellularly and transmit no signals into intracellular proteins based on the transcriptomic profile (no downstream cascade was initiated), so drug target analysis is not appropriate for the SHS-4D vs. mock-treated controls comparison. Regarding the comparison of SHS-6D vs. mock-treated controls, intracellular transcripts are differentially expressed, which justifies the possible transmission of signals and provides an opportunity for identifying drug targets.

In SHS-6D vs. mock-treated controls, we have identified the top drug targets among all the pathways that were enriched by SPIA algorithms (Table 4). Many common drug targets, such as IL6, TP53, and AKT1, appear. Estrogen receptor 1 (*ER1*) is a ligand-activated receptor located at the nuclear membrane, acting as a transcription factor. Tyrosine-protein kinase *KIT* is a cytokine receptor that influences cell survival, proliferation, and differentiation by activating its downstream pathways.

### 3.4. GSEA Pathway Analysis

To support our SPIA enrichment results, we additionally applied GSEA enrichment with pathway databases KEGG and Reactome (Table 5). GSEA tends to generate a greater number of enrichment results and may overlook genes with moderate differential expression. However, we still found consistency between the GSEA and SPIA results. In SHS-4D vs. mock-treated control, we once again discovered the association with the complement cascade, and in SHS-6D vs. mock-treated control, we see overwhelming enrichment results on associations with collagen regulation/ modification pathways. It is essential to note that, based solely on GSEA results, one cannot determine the activation/deactivation of a pathway. The complete GSEA enrichment data are provided [Supplementary File].

### 3.5. Literature Associations with DEGs

**Figure 4:**
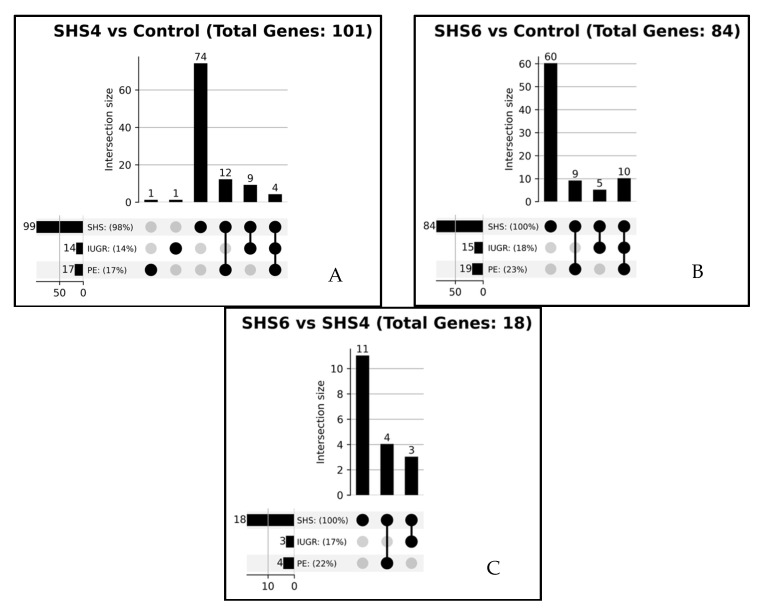
Upset Graphs of Gene-Disease Associations Across SHS Datasets with Reference Threshold > 0. Each graph shows the number of genes in each intersection (bar labels) and the percentage of total genes for each condition. The grey dots un the lower panel represent the groups that overlapped. (**A**) SHS-4D vs. mock-treated control, (**B**) SHS-6D vs. mock-treated control, (**C**) SHS-6D vs. SHS-4D.

To contextualize DEGs within existing knowledge and test the relevance of hypotheses, we analyzed literature associations for SHS, PE, and IUGR. In order to identify genes specifically associated with SHS-4D-induced IUGR and SHS-6D-induced PE, we constructed the following queries: “*SHS AND PE AND IUGR AND DEGs (from each group)*” in the PubTator3 API (Figure 4). Among DEGs from the three comparisons, we found that almost all DEGs are associated with SHS (SHS-4D vs. mock-treated controls: 99% of DEGs are associated with SHS; SHS-6D vs. mock-treated controls: 100%; SHS-6D vs. SHS-4D: 100%). In the SHS-4D-induced IUGR model, we identified four genes (*EGFR*, *MUC16*, *PROZ*, *PTGS2*) that were co-mentioned in publications discussing all three conditions: SHS, PE, and IUGR. EGFR, epidermal growth factor receptor, regulates cell proliferation. Similarly, in the SHS-6D-induced PE model, ten genes (*COL1A1*, *COL17A1*, *EDN2*, *CUL7*, *HDAC4*, *MUC16*, *NOS1*, *PTGS2*, *TET3*, and *VEGFA*) are referenced in papers mentioning all three terms. Notably, collagen-associated genes (*COL1A1*, *COL17A1*), along with *EDN2* and *NOS1*, which regulate vasoconstriction, and *CUL7*, a protein aiding E3 ubiquitin ligase assembly, are well-documented in PE pathogenesis. The high SHS association (99–100% of DEGs) with limited PE/IUGR overlap supports the novelty of our temporal discoveries in addressing understudied SHS-induced pathways.

### 3.6. Immune Infiltration Analysis

**Figure 5:**
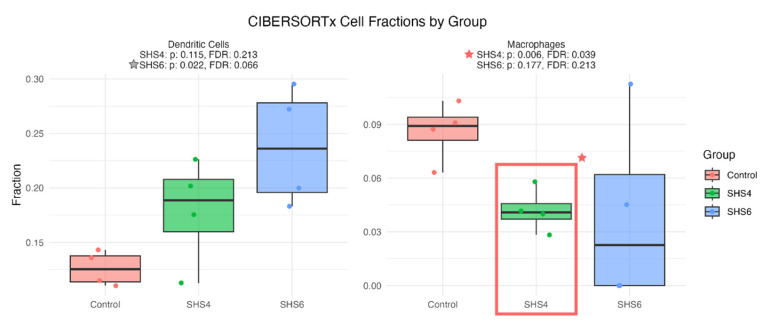
CibersortX immune filtration analysis on changes in immune cell-type population with Control (salmon), SHS-4D (green), and SHS-6D (blue) groups across two cell types—dendritic cells (**left**)and macrophages (**right**). The *p*-values were FDR-adjusted. Cell types and statistics are shown in the titles of each box plot. The red box with a star in the right panel indicates statistically significant changes in the fraction of macrophages in the SHS-4D group compared to the Control group.

Immune deconvolution (CIBERSORTx) tests the immune component of the hypothesis by quantifying cell-type signals. To investigate the role of immune cells in the temporal effects of SHS on pregnant mice, we performed an immune infiltration analysis to detect changes in immune cell signals (Figure 5). In the SHS-4D-induced IUGR model, there were significantly fewer signals for macrophage cells, aligning with our previous observations from the pathway analyses, suggesting a weakened innate immune response. On the other hand, in the SHS-6D-induced PE model, although not statistically significant after FDR adjustment, there is a potential increase in the fraction of dendritic cells. Reduced macrophage signals in SHS-4D (FDR-adjusted *p*-value significant) align with complement weakening in IUGR, while potential dendritic increases in SHS-6D suggest immune shifts in PE, showing distinct temporal responses.

## 4. Discussion

This study shows the biological processes underlying SHS-induced placental dysfunction, contributing to adverse pregnancy outcomes such as PE and IUGR in a C57BL/6 mouse model. Through bulk RNA-sequencing, we identified 88 significant DEGs in placental tissues exposed to SHS, revealing that the timing of exposure relative to SA invasion (E14.5 to E18.5 for SHS-4D inducing IUGR-like symptoms vs. E12.5 to E18.5 for SHS-6D inducing PE-like symptoms) activates distinct biological pathways. Specifically, SHS exposure after spiral artery invasion (SHS-4D) is associated with IUGR-like symptoms along with inhibition of complement system pathways. In contrast, exposure before invasion (SHS-6D) is associated with PE-like symptoms along with inhibition of collagen formation pathways.

### 4.1. SHS Exposure and IUGR: Complement System and Immune Dysregulation

SHS exposure after spiral artery invasion (E14.5, SHS-4D) results in IUGR, as demonstrated by a significant reduction in both placental and fetal weights. Pathway analysis with SPIA and GSEA both suggested inhibition of the complement system, with downregulated genes such as *C1QA* and *OLR1*.

SHS-induced complement suppression may also contribute to placental inflammation. This pro-inflammatory state aligns with reports that SHS increases levels of cytokines such as *IL-4*, *IL-5*, and *TNF-α* in human plasma, which persist for even hours after exposure [41]. Recent studies further corroborate these mechanisms. For instance, SHS exposure was associated with increased risks of low birth weight and preterm delivery, outcomes linked to placental insufficiency and complement pathway alterations [41]. Similarly, passive smoking during pregnancy is significantly associated with adverse birth outcomes, including reduced fetal growth [42], potentially mediated by inflammatory responses. In a murine model, antenatal SHS exposure has been shown to alter metabolic programming via the receptor for advanced glycation end-products (RAGE), leading to long-term organ dysfunction that aligns with our observed complement downregulation and reduced macrophage infiltration [42]. These findings provide a supportive context for our results, where SHS-4D exposure after SA invasion inhibits complement genes, such as *C1QA*, contributing to IUGR-like reductions in fetal weight. The novelty of our work lies in pinpointing timing-specific transcriptomic shifts.

Additionally, SHS inhibits placental mTOR signaling, a key regulator of nutrient transport and trophoblast invasion, further contributing to IUGR [43]. Genes such as *NPTX2* and *KPNA2*, which were downregulated in the SHS-4D dataset, are associated with impaired nutrient transport and placental growth, which provides support for this mechanism. mTOR activity has been positively correlated with *KPNA2* activity and is suggested to regulate the transcriptional and post-translational expression of *KPNA2* via proteasomal degradation, as well as the expression of *DP1* and *E2F1* [44]. The combined effects of complement dysregulation, inflammation, and mTOR inhibition likely exacerbate placental insufficiency, reducing fetal nutrient delivery and growth.

### 4.2. SHS Exposure and Preeclampsia: Collagen Formation and ECM Dysregulation

Exposure to SHS before SA invasion (E12.5, SHS-6D) induced PE-like symptoms, including significantly elevated systolic and diastolic blood pressure, as well as increased proteinuria, after six days of exposure. RNA-sequencing analysis revealed significant downregulation of collagen-related genes, such as *COL4A5* and *COL15A1*, within pathways inhibited by collagen formation. The placental ECM, composed of collagen types I, III, IV, and V, is essential for supporting trophoblast invasion and SA remodeling, which are critical for adequate placental perfusion [45]. In human PE, excessive collagen deposition increases placental tissue rigidity, impairing trophoblast function and contributing to shallow invasion and poor placentation [46]. However, our results contradict this by showing collagen downregulation, which may represent an alternative mechanism in SHS-induced PE in this model system. Notably, studies on direct maternal smoking have reported increased collagen deposition in human placentas, suggesting that SHS may differentially affect ECM remodeling compared to active smoking, potentially due to lower toxin doses or indirect exposure routes [45,47].

### 4.3. Timing of Exposure and Pathway-Specific Effects

The minimal overlap between PE- and IUGR-associated literature among all DEGs in this study suggests that the SHS-induced PE mouse model is strongly associated with SHS exposure but not with PE itself. At the same time, our findings indicate that SHS exposure during early and late gestation separately initiates distinct PE pathogenesis while ultimately developing converging symptoms, adding another layer of perplexity to our specific phenotypes.

### 4.4. Limitations and Future Directions

While our mouse model provides valuable mechanistic insights, differences in placental structure and gestation between mice and humans limit direct translatability. The nose-only exposure system, while controlled, may not fully replicate the chronic, low-level SHS exposure experienced by humans in real-world settings. Additionally, bulk RNA-sequencing may at least partially mask cell-specific effects within the heterogeneous placental tissue. Future studies using single-cell RNA sequencing will show cell-type-specific responses, such as those in trophoblasts or endothelial cells, or cell-region responses, such as those in the junctional zone or the labyrinth, to SHS exposure [48]. For example, scRNA-seq has revealed distinct molecular profiles in early- and late-onset PE [49], including cellular senescence in placental mesenchymal cells [50]. and transcriptional heterogeneity in trophoblasts [51]. Applying similar techniques to SHS models could identify targeted pathways for intervention. Integrating proteomic and metabolomic analyses could further clarify the downstream effects of DEGs, providing a more comprehensive understanding of SHS-induced placental dysfunction. Longitudinal human cohort studies are needed to validate our findings, particularly in populations with high SHS exposure. These studies should explore the dose–response relationship of SHS and assess biomarkers of placental function, such as *MUC16* or *C1QA*, in maternal blood or placental tissue.

## 5. Conclusions

This study demonstrates that SHS exposure during pregnancy disrupts placental biology in a timing-dependent manner, with pre-invasion exposure (SHS-6D) driving PE-like symptoms through inhibition of the collagen pathway and post-invasion exposure (SHS-4D) contributing to IUGR via dysregulation of the complement system. These findings provide critical mechanistic insights into SHS-induced pregnancy complications and highlight the importance of public health strategies to reduce SHS exposure in pregnant populations. By elucidating the molecular pathways affected by SHS, our work lays the groundwork for targeted interventions to enhance maternal and neonatal health outcomes worldwide.

## Figures and Tables

**Table 1:** The top five up- and down-regulated differentially expressed genes were detected in the placental tissues compared to SHS-4D vs. mock-treated controls. Sorted by decreasing log_2_ fold-change values.

Symbol	Description	Log_2_FC	FDR
IL11RA3	interleukin 11 receptor subunit alpha	8.0	0.003
ECE2	endothelin converting enzyme 2	4.7	0.016
CHIL3	chitinase acidic	2.5	<0.001
KPNA2	karyopherin subunit alpha 2	2.1	0.014
NPTX2	neuronal pentraxin 2	1.9	<0.001
UPK3BL	uroplakin 3B like 1	−4.0	<0.001
ARHGAP40	Rho GTPase-activating protein 40	−4.1	<0.001
SLCO1B2	solute carrier organic anion transporter family member 1B3	−4.6	0.002
KRT15	keratin 15	−4.8	0.002
UGT1A9	UDP glucuronosyltransferase family one member A9	−5.2	0.03

**Table 2:** The top five up- and down-regulated differentially expressed genes detected in the placental tissues in the comparison of SHS-6D vs. mock-treated controls. Sorted by decreasing log_2_ fold-change values.

Symbol	Description	Log_2_FC	FDR
TPSAB1	tryptase alpha/beta 1	9.8	0.001
PLCXD1	phosphatidylinositol-specific phospholipase C X domain-containing 1	2.2	0.023
RTP3	receptor transporter protein 3	1.8	0.035
CD6	CD6 molecule	1.8	0.029
EDN2	endothelin 2	1.8	0.007
UPK3BL	uroplakin 3B like 1	−2.9	0.008
PRSS27	serine protease 27	−3.0	0.04
CLEC3B	C-type lectin domain family three member B	−3.1	0.020
MUC16	mucin 16, cell surface-associated	−3.2	0.037
KRT5	keratin 5	−3.3	0.007

**Table 3:** The top five up- and down-regulated differentially expressed genes detected in the placental tissues from the SHS-6D vs. SHS-4D comparison. Sorted by decreasing log_2_ fold-change values.

Symbol	Description	Log_2_FC	FDR
TPSAB1	tryptase alpha/beta 1	10.0	0.001
PPIHL	peptidylprolyl isomerase H	8.0	0.036
GZMB	granzyme B	4.6	<0.001
GZMD	granzyme D	4.1	<0.001
PRF1	perforin 1	3.8	0.007
IRX5	Iroquois homeobox 5	−1.6	0.027
XRRA1	X-ray radiation resistance associated with 1	−1.7	0.036
NPTX2	neuronal pentraxin 2	−1.7	<0.001
TBX1	T-box transcription factor 1	−1.8	0.018
RGS9	regulator of G protein signaling 9	−2.3	0.001

**Table 4:** Top nine drug targets for the SPIA-enriched pathways in the SHS-6D vs. mock-treated control comparison. The columns include the gene symbols, subcellular location, the number of times such a target appears in all the pathways (target in pathways), the number of drugs targeting such a gene that are FDA approved, and the number of drugs in clinical trial phases 1~4.

Target Symbol	Subcellular Location	Number Pathways with Target	Number Approved Drugs	Number Phase 1	Number Phase 2	Number Phase 3	Number Phase 4
IL6	Secreted	7	1	0	1	3	1
TP53	Cytoplasm	8	0	0	3	2	0
EGFR	Cell membrane	9	5	0	0	0	5
ESR1	Nucleus	6	5	0	0	0	5
AKT1	Cytoplasm	9	1	0	3	1	1
MTOR	Lysosome	6	1	0	1	3	1
PIK3CA	Cytosol	9	3	0	0	2	3
KIT	Cell membrane	6	5	0	0	0	5
PTGS2	Microsome	8	5	0	0	0	5

**Table 5:** GSEA Pathway Enrichment Results Among SHS-4D vs. mock-treated control (SHS-4D) and SHS-6D vs. mock-treated control (SHS-6D). The table contains selected results from GSEA enrichment on the KEGG and Reactome pathway databases. The *q*-values are filtered with a value below 0.05. Description columns showing the name of the pathways, and the comparison columns showing the comparison groups.

Description	Adjusted *p*-Value	*q*-Value	Comparison
Complement and coagulation cascades	9.14 × 10^−3^	7.54 × 10^−3^	SHS-4D
Immunoregulatory interactions between a Lymphoid and a non-Lymphoid cell	1.17 × 10^−3^	1.07 × 10^−3^	SHS-4D
Complement cascade	2.36 × 10^−3^	2.16 × 10^−3^	SHS-4D
Collagen formation	2.03 × 10^−8^	1.66 × 10^−8^	SHS-6D
Collagen biosynthesis and modifying enzymes	2.03 × 10^−8^	1.66 × 10^−8^	SHS-6D
Assembly of collagen fibrils and other multimeric structures	2.03 × 10^−8^	1.66 × 10^−8^	SHS-6D
Collagen degradation	2.03 × 10^−8^	1.66 × 10^−8^	SHS-6D
Collagen chain trimerization	2.03 × 10^−8^	1.66 × 10^−8^	SHS-6D
Extracellular matrix organization	2.03 × 10^−8^	1.66 × 10^−8^	SHS-6D
ECM proteoglycans	7.27 × 10^−8^	5.95 × 10^−8^	SHS-6D
Degradation of the extracellular matrix	3.28 × 10^−7^	2.68 × 10^−7^	SHS-6D
Crosslinking of collagen fibrils	1.79 × 10^−6^	1.47 × 10^−6^	SHS-6D
Integrin cell surface interactions	4.82 × 10^−5^	3.95 × 10^−5^	SHS-6D
ECM-receptor interaction	3.61 × 10^−4^	3.13 × 10^−4^	SHS-6D
Focal adhesion	9.29 × 10^−3^	8.05 × 10^−3^	SHS-6D

## Data Availability

The original contributions presented in the study are included in the article. Pre-processed reads are available at https://zenodo.org/records/16825230 (accessed on 25 August 2025).

## Supplementary Materials

The following supporting information can be downloaded at: https://www.mdpi.com/article/10.3390/cells14211735/s1.
